# Efficacy of a topical formulation containing MIA (Melanoma Inhibitory Activity) ‐ Inhibitory peptides in a case of recalcitrant vitiligo in combination with UV exposure

**DOI:** 10.1097/MD.0000000000031833

**Published:** 2022-11-18

**Authors:** Sergi Hernandez Navarro, Jordi Segura Tejedor, Marta Bajona Roig, Roberto Luisetto, Marny Fedrigo, Chiara Castellani, Annalisa Angelini, Mauro Alaibac, Matteo Bordignon

**Affiliations:** a R&D Department, Bella Aurora Labs, Barcelona, Spain; b Department of Surgical Oncological and Gastroenterological Sciences, University of Padua, Padova, Italy; c Department of Cardiac Thoracic and Vascular Sciences, University of Padua, Padova, Italy; d Unit of Dermatology, University of Padua, Padova, Italy.

**Keywords:** vitiligo, melanoma inhibitory activity, therapy, MIA inhibitors

## Abstract

Vitiligo is an acquired chronic pigmentation disorder of the skin. Even if the role of the immune system seems to be well established, new pathogenetic hypothesis are rising in these years. It has been recently suggested by the development of an animal model that a protein called Melanoma Inhibitory Activity (MIA) is involved in the pathogenesis of vitiligo. This protein interacts with the adhesion molecules expressed on the melanocytes causing its detachment from extracellular matrix proteins and creating the depigmented macules. A topical preparation based on oligopeptides able to inhibit the actions of the MIA protein has been introduced to the market, claiming activity on vitiligo.

**Patient concerns and diagnosis:**

A patient affected by non-segmental vitiligo for 10 years, recalcitrant to any treatment (such as steroids, immunomodulators, kellin, UVB-NB and UVA) came to our observation.

**Interventions:**

We used this topical preparation containing the MIA inhibitors peptides in selected areas (face and sides of the trunk) leaving untreated other areas as control (legs and arms). The patient was required to be sun exposed or to have some UVA sessions during the treatment to stimulate the melanocytes replications.

**Outcomes:**

After 9 months of treatments, he recovered from 50% to 80% of repigmentation only in the treated areas, without any side effects locally or systemically.

**Conclusion:**

Even if other studies are required to better determine the efficacy of this approach, this first observation about the use of the MIA-inhibitors peptides for the treatment of non-segmental vitiligo indicates that this topical preparation containing the MIA inhibitors peptides could be a very promising option for the cure of this disease.

## 1. Introduction

Vitiligo is an acquired chronic pigmentation disorder of the skin that affects 0.5–2% of the population worldwide.^[1]^ The exact pathogenesis of the dermatosis is still to be fully clarified.

**Figure 1.**
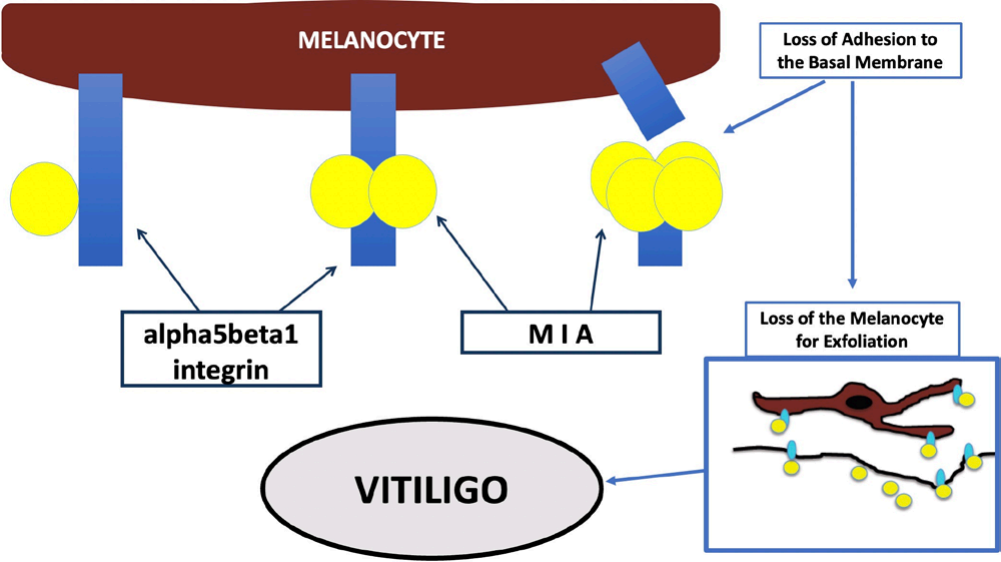
Scheme of the pathogenesis of vitiligo induced by MIA (Melanoma Inhibitory Activity). The adhesion molecules of the melanocytes (particularly the alpha5beta1 integrins) are bind by MIA in its dimeric and tetrameric forms (which are the active species). The breaking of the integrins induces the loss of adhesion of the melanocytes to the basal membrane and its exfoliation for melanocytorrhagy, leading to the formation of the achromic patches on the skin surface.

Even if the main role of the immune system seems to be well established, it was previously suggested that at least the final step of the formation of the achromic patches could be mediated by a different mechanism, such as the action of a protein called Melanoma Inhibitory Activity (MIA).^[2]^ MIA is a small protein firstly described as secreted from malignant melanoma cells, which is able to interact with a particular group of adhesion molecules called alpha5beta1 integrins (a5b1ints).^[3]^ This protein has been found present also in skin of vitiligo-affected patients, interacting with the same adhesion molecules.^[2]^ The binding of MIA to these proteins at the cell surface is responsible for the detachment of melanocytes from extracellular matrix proteins, creating the depigmented macules through melanocytorrhagy, as recently demonstrate also in an animal model^[4]^ (Fig. 1). This process is consistent with the absence of an inflammatory response clinically evident on the vitiligo-affected skin and with the clear-cut edge of the lesions observed clinically and histologically. According to this hypothesis, melanocytes are perfectly functional and they are detached singularly and silently by MIA, in accordance with the skin appearance of vitiligo-affected patients.^[4]^

Considering all these evidences, the MIA protein could surely be a very interesting target for treating patients affected by vitiligo, since the inhibition of this molecule would leave the melanocytes unperturbed and able to replicate more easily to recover the skin color.

**Figure 2.**
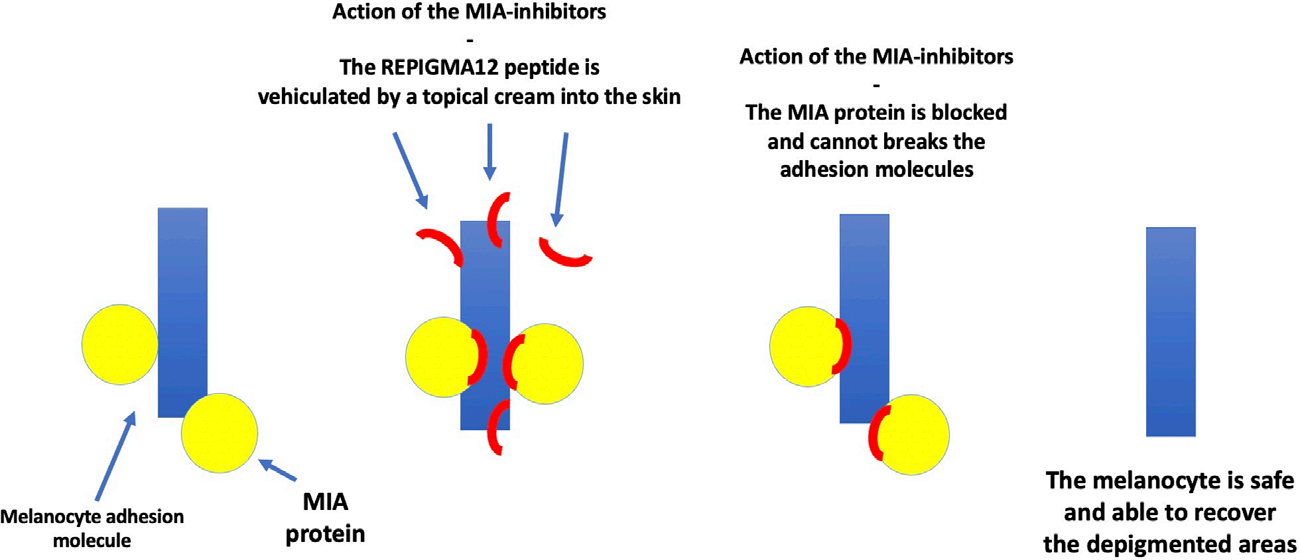
Action of the MIA-inhibitors. The topical preparation containing the MIA-inhibitors properly vehiculates these peptides through the skin toward the MIA protein itself. The peptides are able to bind the MIA protein to its dimerization site, avoiding the formation of the active species and thus avoiding the breakage of the melanocyte’s adhesion system to the basal membrane. In this way, the melanocyte is stable and able to recover the depigmented areas through replication.

Due to its peculiar mechanism of action, the use of inhibitory peptides is surely a very interesting approach to block the MIA protein through a topical delivery system. The single units of the MIA proteins are inactive whereas the active form is made by dimers or tetramers of these species.^[5]^ Thus, the inhibition strategy of the MIA protein consists in using a particular sequence of 12 amino acids able to bind the protein in its dimerization site avoiding the creation of the active forms (Fig. 2).

Recently, a topical preparation based on these oligopeptides able to inhibit the MIA protein has been introduced to the market, claiming activity on vitiligo.

In this study we describe a clinical case of recalcitrant vitiligo successfully treated with this topical preparation in combination with UV exposure.

## 2. Case report

A 50 years old male patient, affected by non-segmental vitiligo for 10 years, came to our observation. During the years, he was treated with various therapies including oral and topical steroids, topical immunomodulators (tacrolimus and pimecrolimus), kellin, UVB-NB and UVA exposure, without any benefit and with progressive enlargement of the white spots.

**Figure 3.**
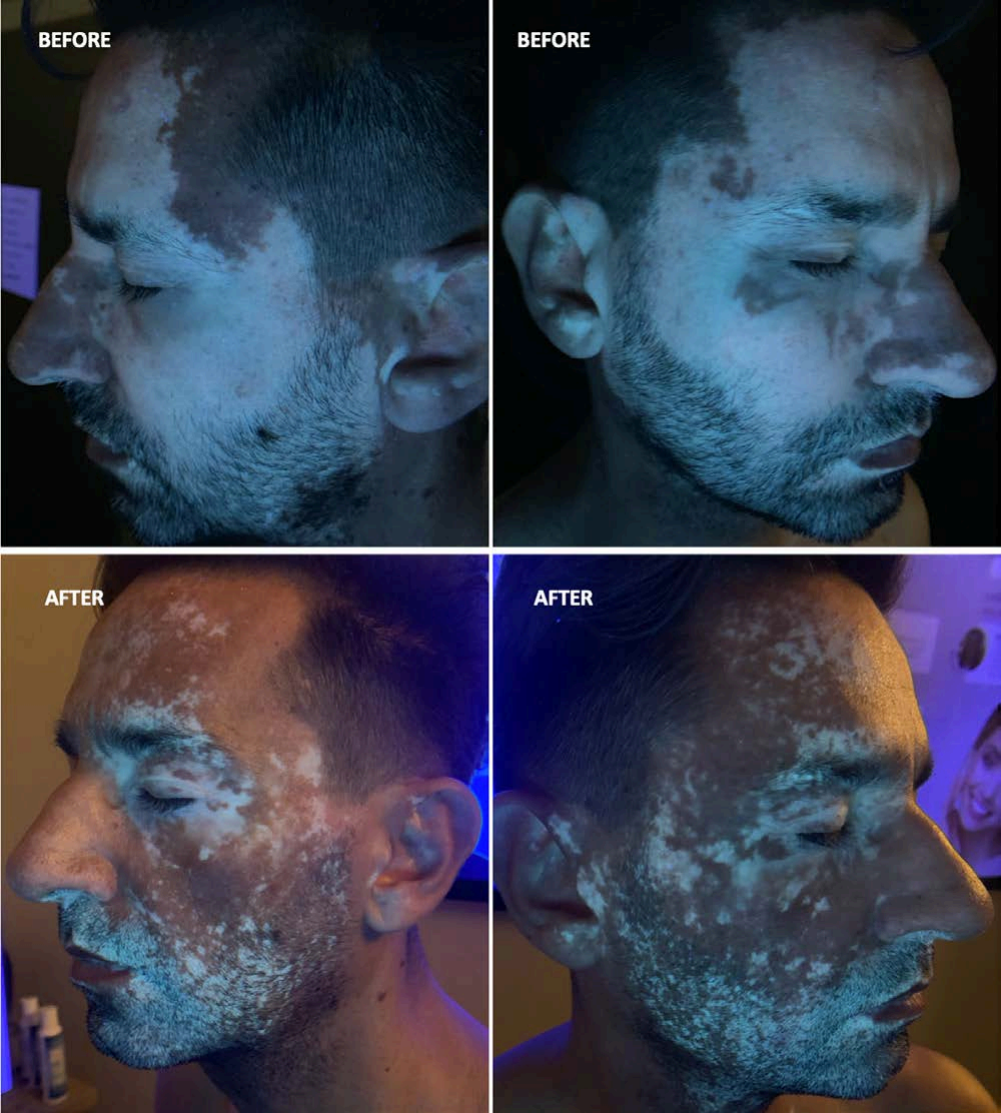
Patient treated with cosmetic preparation of oligopeptides inhibiting the MIA protein, together with sun or UVA (sunbeds) exposure and oral antioxidants. After 9 months of treatment, the repigmentation achieved was about 80% of baseline.

**Figure 4.**
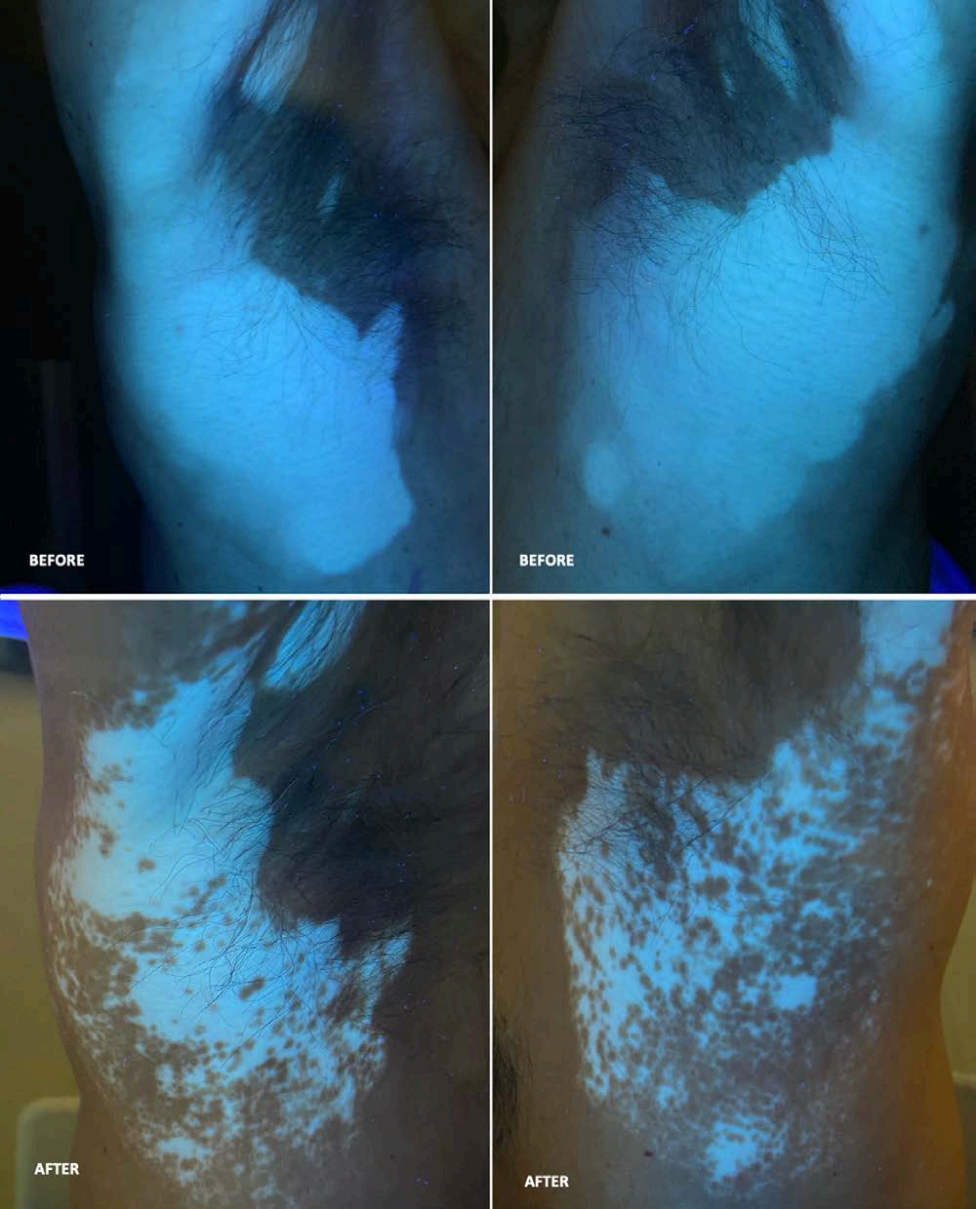
Patient treated with cosmetic preparation of oligopeptides inhibiting the MIA protein, together with sun or UVA (sunbeds) exposure and oral antioxidants. After 9 months of treatment, the repigmentation achieved was about 50% of baseline on the right side and 70% of baseline on the left side.

The spots were localized mainly in the face (about 90% of depigmentation—Fig. 3—Before Panels) and lateral sides of the trunk (Fig. 4—Before Panels) but also on hands, feet, arms and legs.

The patient did never experience spontaneous repigmentation of any lesions during the sun exposure and he was just assuming oral antioxidants in the last 12 months for avoiding sun damage of the skin.

After full explanation, taking a personal consent of the patient and agreement on regular follow up, we decided to use the cosmetic topical treatment based on the MIA-inhibitors technology (Repigment12^TM^ cream, Bella Aurora Labs, Barcelona, Spain).

The topical treatment was applied twice a day for 9 months on the affected skin of the face and trunk and the patient was monitored on a monthly basis to verify the presence of repigmentation of the skin and/or any possible onset of side effects (local and systemic). The other parts affected by vitiligo were left untreated because of the well-known difficulty to achieve any result (hands and feet) or as control to evaluate the role of UV exposure (legs and arms). The patient was asked to be sun-exposed daily or to have some session of UVA lamps (18 sessions totally in 4 months performed) in order to have melanocytes stimulation. The patient was forbidden to perform UVB-NB in order to avoid possible bias in interpreting the results of the treatment and asked to continue the antioxidants assumption.

During the months, the repigmentation started to be restored only in the application sites (face and sides of the trunk) after 3 months of treatment and after 9 months of observation it reached from the 50% to 80% of the basal situation (see Figs. 3 and 4–“After” Panels). The untreated parts affected by vitiligo remained as they were at the starting of our observation.

During the treatment, no side effects locally or systemically of any type were observed.

## 3. Discussion and conclusion

Melanocytorrhagy induced by the MIA protein is a new and very interesting pathogenetic hypothesis for vitiligo,^[2,4,6]^ particularly for the possibility to have new targeted treatment for this condition. So far, there were no available treatments in the market designed to inhibit this protein in order to improve the vitiligo spots. Recently, a cosmetic treatment based on the MIA-inhibitor technology (an oligopeptide specifically designed to block the activity of the protein) was available for the topical treatment of white macules.

The technology of the inhibitory peptides is a very interesting tool for the treatment of skin condition because, through the proper vehiculation, these peptides could be very targeted and effective with poor whereas completely absent side effects. This kind of approach is surely very complicated to develop due to the fact that it is required to find a specific aminoacidic sequence interacting with a specific part of the protein to be inhibited. Once the right sequence is found, anyway, the benefits are very important because the peptide interacts with its target or otherwise it is simply degraded into basic amino acid giving a very elevated safety profile of the treatment, like reported in previous studies.^[7]^

Regarding the peptides able to block the MIA protein, they are a series of different chains of amino acids with the property to interact with the protein itself. Among these, one particular sequence of 12 amino acids (the so called Repigma12 peptide) is able to bind the MIA protein at its dimerization site, avoiding in this manner the formation of the dimeric or tetrameric active form.

We use this new treatment in case of recalcitrant and very diffuse non segmental vitiligo, in combination with sun or UVA exposure and oral antioxidants with very interesting results. The treatment was able to restore up to 80% of the pigmentation of the white patches, without any side effects locally or systemically. We use the treatment in combination with sun or UVA exposure because of the necessity of stimulate the replication and activity of the melanocytes once the activity of the MIA protein was blocked by the peptides present in the topical treatment. Antioxidants, moreover, were used to reduce the possible side effects of the UV exposure on the skin.

The role of the MIA-inhibitors seems to be very decisive for achieving the clinical improvement in this patient. In fact, the patient was already subjected in the past to UV or sun exposure without any results and at the same way he was using antioxidants in the last 12 months, without any sign of improvement. Moreover, the part not treated with the MIA-inhibitors cream but anyway benefited by UV or sun exposure and the action of antioxidants did not show any sign of repigmentation.

From a safety point of view, very interestingly the treatment did not show any side effects, both topically and systemically. The use of the MIA-inhibitors in this patient involved very diffused spots for a prolonged amount of time, without the appearance of any undesired effects. This observation is very promising for the use of this kind of approach in the vitiligo affected patients as it is well known that this kind of patients required a long period of treatment in often very wide patches diffused in all the body. To be still determined anyway is the theoretical risk of sensitization and if the same safety profile (particularly from a topical point of view) is maintained also for more thin part of the skin such as eyelids.

In conclusion, this case report is the first observation of a successful treatment of non-segmental vitiligo using the MIA-inhibitors technology. Based on the very interesting results reported, this treatment could be considered a possible therapeutic option of non-segmental vitiligo. Other studies are mandatory to establish the real efficacy of this treatment with or without UV exposure and to determine the exact safety profile of this approach (which seems to be very elevated) together with the efficacy in other and more resistant areas such as hands and feet.

## Acknowledgments

The authors thank Dr A.P. De Cata, Mr. A.E.D. Bordignon and Mr J. M. Martinez Ribes for supporting their work.

## Author contributions

**Conceptualization:** Jordi Segura Tejedor, Matteo Bordignon, Sergi Hernandez Navarro.

**Data curation:** Annalisa Angelini, Chiara Castellani, Jordi Segura Tejedor, Matteo Bordignon, Marny Fedrigo, Marta Bajona Roig, Roberto Luisetto, Sergi Hernandez Navarro.

**Formal analysis:** Matteo Bordignon.

**Investigation:** Matteo Bordignon.

**Supervision:** Mauro Alaibac, Matteo Bordignon.

**Validation:** Mauro Alaibac, Matteo Bordignon.

**Writing – original draft:** Jordi Segura Tejedor, Marta Bajona Roig, Matteo Bordignon, Sergi Hernandez Navarro.

**Writing – review & editing:** Annalisa Angelini, Chiara Castellani, Jordi Segura Tejedor, Marta Bajona Roig, Matteo Bordignon, Mauro Alaibac, Roberto Luisetto, Sergi Hernandez Navarro.
